# Modulation of B-cell endoplasmic reticulum calcium homeostasis by Epstein-Barr virus Latent Membrane Protein-1

**DOI:** 10.1186/1476-4598-8-59

**Published:** 2009-08-03

**Authors:** Olivier Dellis, Atousa Arbabian, Jean-Philippe Brouland, Tünde Kovàcs, Martin Rowe, Christine Chomienne, Irène Joab, Béla Papp

**Affiliations:** 1 Institut National de la Santé et de la Recherche Médicale, Inserm UMR-S 940, Institut Universitaire d'Hématologie, Université Paris VII, 16, rue de la Grange aux Belles, 75010 Paris, France; 2 Service d'Anatomie et Cytologie Pathologiques, Hôpital Lariboisière, 2, rue Ambroise Paré, 75010 Paris, France; 3 Membrane Research Group, Hungarian Academy of Sciences, Diószegi u. 64, H-1113 Budapest, Hungary; 4 Division of Cancer Studies, University of Birmingham Medical School, Edgbaston B15 2TT, UK; 5 Institut National de la Santé et de la Recherche Médicale, Inserm UMR542-Université Paris Sud, Hôpital Paul Brousse, Paris, France

## Abstract

**Background:**

Calcium signaling plays an important role in B lymphocyte survival and activation, and is critically dependent on the inositol-1,4,5-*tris*-phosphate-induced release of calcium stored in the endoplasmic reticulum (ER). Calcium is accumulated in the ER by Sarco/Endoplasmic Reticulum Calcium ATPases (SERCA enzymes), and therefore these enzymes play an important role in ER calcium homeostasis and in the control of B of cell activation. Because Epstein-Barr virus (EBV) can immortalize B cells and contributes to lymphomagenesis, in this work the effects of the virus on SERCA-type calcium pump expression and calcium accumulation in the endoplasmic reticulum of B cells was investigated.

**Results:**

Two Sarco-Endoplasmic Reticulum Calcium transport ATPase isoforms, the low Ca^2+^-affinity SERCA3, and the high Ca^2+^-affinity SERCA2 enzymes are simultaneously expressed in B cells. Latency type III infection of Burkitt's lymphoma cell lines with immortalization-competent virus expressing the full set of latency genes selectively decreased the expression of SERCA3 protein, whereas infection with immortalization-deficient virus that does not express the EBNA2 or LMP-1 viral genes was without effect. Down-modulation of SERCA3 expression could be observed upon LMP-1, but not EBNA2 expression in cells carrying inducible transgenes, and LMP-1 expression was associated with enhanced resting cytosolic calcium levels and increased calcium storage in the endoplasmic reticulum. Similarly to virus-induced B cell immortalisation, SERCA3 expression was also decreased in normal B cells undergoing activation and blastic transformation in germinal centers of lymph node follicles.

**Conclusion:**

The data presented in this work indicate that EBV-induced immortalization leads to the remodelling of ER calcium homeostasis of B cells by LMP-1 that copies a previously unknown normal phenomenon taking place during antigen driven B cell activation. The functional remodelling of ER calcium homeostasis by down-regulation of SERCA3 expression constitutes a previously unknown mechanism involved in EBV-induced B cell immortalisation.

## Background

Endoplasmic reticulum (ER) calcium homeostasis plays an important role in the control of cell activation [1,2]. Calcium release from the ER by the second messenger D-*myo*-inositol-1,4,5-*tris-*phosphate (IP3) upon activation of receptors such as the B-cell or chemokine receptors, combined with ensuing calcium influx across the plasma membrane (store operated calcium entry, SOCE), leads to the activation of key calcium-dependent enzymes involved in cell activation such as various protein kinase C isoforms, calcineurin or calmodulin dependent kinases [2-6]. Because these enzymes critically modulate the activity of transcription factors such as NF-κB, NF-AT or AP1, calcium signalling, initiated by calcium release from the ER, plays important roles in B cell survival, maturation and activation. In addition, normal ER calcium homeostasis is also required for the post-translational modifications/chaperoning of nascent immunoglobulin molecules taking place in the organelle, a phenomenon that is also involved in the regulation of B cell maturation and plasma cell survival [7-9].

Because calcium accumulation into the endoplasmic reticulum (ER) is accomplished by Sarco/Endoplasmic Reticulum Calcium ATPases (SERCA-type calcium pumps) that generate a strong calcium concentration gradient between the cytosol (low nanomolar) and the ER lumen (high micromolar), precisely regulated SERCA activity is essential for normal cell function and survival [10]. SERCA-type calcium pumps are encoded by three genes (SERCA1, 2 and 3) that give rise by alternative splicing to several isoenzymes. Whereas SERCA1 is expressed in skeletal muscle, and the SERCA2a isoform is found in cardiac muscle, SERCA2b expression is ubiquitous. In several cell types such as T lymphocytes, myeloid cells, megakaryocytes and platelets, as well as in colon and gastric epithelial cells SERCA2b is co-expressed with SERCA3 [11-18]. Whereas the basic biochemical function (*i.e*. calcium transport into the ER) is shared among SERCA2 and SERCA3 isoenzymes, the calcium affinity of SERCA3 is significantly inferior to that of SERCA2b [10,19-22]. The co-expression of SERCA2b and SERCA3 within the same cell is thus involved in the fine regulation of the calcium uptake characteristics of the ER in a cell type-dependent manner.

Epstein-Barr virus, a human gammaherpesvirus can immortalize primary naïve B lymphocytes, leading to the establishment of permanently growing lymphoblastoid cell lines (LCL). In addition, the virus is etiologically involved in the formation of several types of malignancies such as Burkitt's lymphoma, lymphomas of immunocompromised individuals, some NK/T lymphomas, lymphomatoid granulomatosis, pyothorax-associated lymphoma, Hodgkin's lymphoma, as well as gastric and nasopharyngeal carcinomas (for a comprehensive review of EBV biology see [23]). EBV-induced immortalization of B cells into LCLs, as well as malignant transformation are linked to the viral latent membrane protein-1 (LMP-1), the expression of which is under the control of the EBNA2 virus encoded protein. LMP-1 displays great functional homology with activated CD40 normally expressed on B cells [23,24]. LMP-1 constitutively activates a variety of signalling cascades including NF-κB, ERK, JNK, and p38 MAP kinase, as well as the Akt-PI3K pathway, is responsible for the phenotypic changes observed during conversion of B cells into LCLs, and is a *bona fide *transforming oncogene in various experimental systems [23,25].

EBV infection, as well as normal lymphocyte activation have been shown to lead to increased resting cytosolic calcium concentration [26,27]. Calcium release from the ER into the cytosol through IP3 receptor calcium channels is essential for B cell function at various stages of B cell differentiation [27-29]. Calcium-dependent cell activation is initiated by calcium release from the ER through IP3 receptors [1,2], and is greatly amplified by subsequent calcium influx from the extracellular space through Orai1-type store-operated calcium channels that are opened by decreased calcium levels in the ER lumen [4,30]. Because calcium release is critically dependent on ER calcium accumulation by SERCA-type enzymes, and because SERCA activity is instrumental in the shaping of the amplitude, the intensity and the duration of cellular calcium signals [31-33] and therefore of cell activation [34-36], in this work we investigated endoplasmic reticulum calcium pump expression and function in various EBV-infected B cells, in cells that express transgenic key EBV proteins such as LMP-1, and compared their effects to normal B cell activation *in situ*.

## Results

### Decreased SERCA3 expression in stably EBV-infected Burkitt's lymphoma cell lines

**Figure 1:**
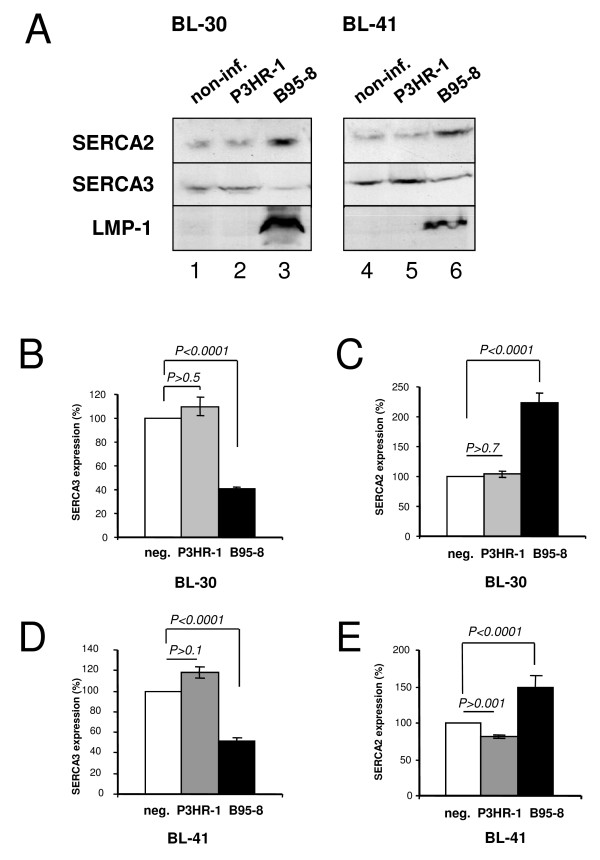
**Decreased SERCA3 expression in Burkitt's lymphoma cell lines infected with immortalization-competent Epstein-Barr virus**. Panel A: Total cellular protein extracts from the BL-30 (lanes 1–3) and BL-41 (lanes 4–6) cell lines free of EBV (lanes 1,4) or chronically infected with the P3HR-1 (lanes 2,5) or the B95–8 (lanes 3,6) EBV strain were analysed by Western blotting for SERCA2 (top) and SERCA3 (center) expression in parallel with the detection of LMP-1 expression (bottom). Panels B,C,D,E: SERCA3 and SERCA2 expression in BL-30 (Panels B and C) and BL-41 cells (Panels D and E) infected with the P3HR-1 (grey columns) or the B95–8 (dark columns) EBV strains, respectively, expressed as % of SERCA expression in non-infected cells (white columns).

BL-30 and BL-41 belong to a set of EBV-negative Burkitt's lymphoma cell lines that have been previously established [37], and subsequently infected with immortalizing, as well as non-immortalizing EBV strains (B95–8 and P3HR-1, respectively) *in vitro*. Infection led to the establishment of latently infected cell lines [38]. Whereas B95–8-infected cells express the whole set of type III latency genes including EBNA2 and LMP-1, P3HR-1-infected cells lack LMP-1 expression due to a deletion in the sequence of EBNA2 that transactivates LMP-1 [38]. In order to investigate the effect of virus on calcium pump expression, equal amounts of total protein extracts from non-infected and infected cells were analysed for SERCA2 and SERCA3 expression in a Western immunoblot format with the IID8 and PLIM430 monoclonal antibodies, respectively. As shown in Fig. 1, infection with the B95–8 virus strain led to decreased SERCA3 expression in both cell lines (Panel A), and similar results were obtained also with BL-2 and BL-31 cells (not shown). As shown in Panels B and D, SERCA3 expression levels in BL-30 and BL-41 cells decreased to 40 +/- 3% (n = 10), and 51 +/- 5% (n = 8) after infection with the B95–8 virus when compared to non-infected cells, whereas SERCA2 expression increased to 223 +/- 35% (n = 6) and 150 +/- 31% (n = 5), respectively. At the same time, infection with the P3HR-1 strain had only marginal effects on the expression of SERCA proteins. Importantly, LMP-1 expression could only be observed in cells stably infected with the B95–8 virus. This is in accordance with earlier observations indicating that LMP-1 expression is absent in P3HR-1-infected cells [38] as a result of lack of EBNA-2.

### Decreased SERCA3 expression during the induction of LMP-1 expression

**Figure 2:**
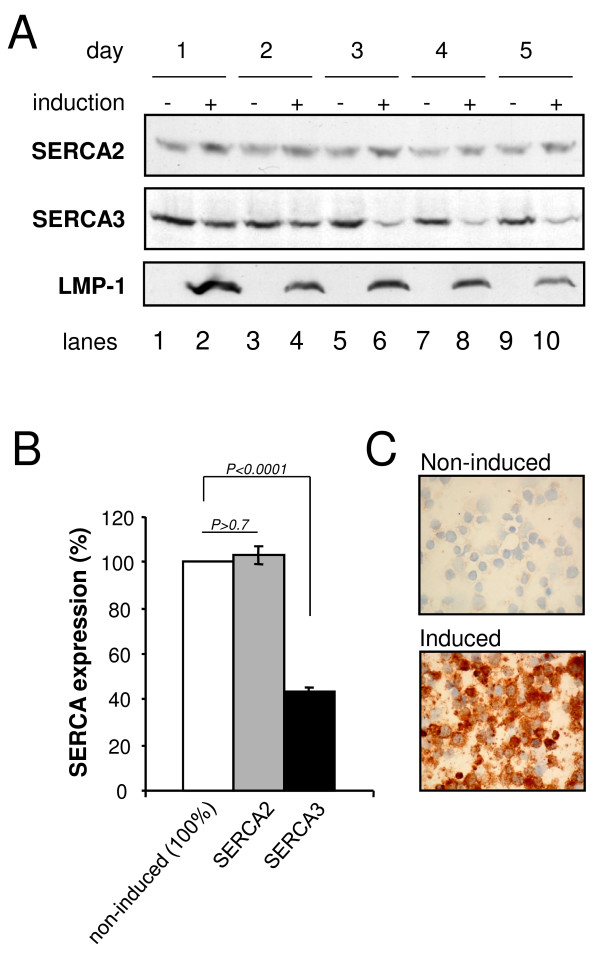
**LMP-1 expression leads to decreased SERCA3 expression**. Panel A: BJAB-tTA-LMP-1 cells were grown in the presence (lanes 1,3,5,7 and 9) or absence (lanes 2,4,6,8 and 10) of 1 μg/ml tetracycline for 5 days, and SERCA2 (top), SERCA3 (center), as well as LMP-1 expression levels (bottom) were determined by Western blotting. Panel B: SERCA 3 (black column) and SERCA2 (grey column) expression in BJAB-tTA-LMP1 cells at day 5 of LMP1 expression. White column: SERCA2 or SERCA3 expression prior induction (100%). Panel C: immunocytochemical detection of LMP-1 expression in BJAB-tTA-LMP1 cells before (top), and after induction (bottom) of LMP-1 expression by tetracycline withdrawal.

Given the association between decreased SERCA3 expression and LMP-1 expression in latently infected cells (Fig. 1), the effect of the direct expression of LMP-1 on SERCA3 expression was investigated using cells stably transfected with inducible vectors coding for LMP-1. The BJAB-tTA-LMP-1 cell line carries an LMP-1 transgene under the control of a tetracycline-repressible promoter. When the cells are grown in the presence of tetracycline, expression of LMP-1 is repressed, and tetracycline withdrawal results in LMP-1 expression at levels similar to that observed in EBV-infected cells [39]. As shown in Fig. 2, tetracycline withdrawal in the BJAB-tTA-LMP-1 cell line resulted in a marked and homogeneous induction of LMP-1 protein as detected by Western blotting (Panel A) and immunocytochemistry (Panel C). Induction of LMP-1 expression led to decreased SERCA3 levels (43 +/- 4.5%, n = 5) during five days (Panels A and B), whereas the expression of SERCA2 was not significantly affected. As a negative control, BJAB-tTA cells, devoid of the LMP-1 transgene were used, and in these cells tetracycline withdrawal had no effect on SERCA expression (not shown). Induction of LMP-1 expression led to decreased SERCA3 expression also in the BL41-MTLM-2 cell line, in which the expression of the LMP-1 cDNA is driven by a heavy metal-inducible promoter derived from the metallothionein gene [40]. When cells were grown in the presence of 100 μM Zn^2+ ^for 3 days, SERCA3 expression was decreased when compared to non-induced controls, similarly to that observed in BJAB-tTA-LMP-1 cells upon tetracycline withdrawal (not shown). It is noteworthy to add, that SERCA3 expression was also investigated in BL41/K3 cells in which the expression of an EBNA2 transgene is driven by an estradiol-inducible promoter [41]. However, induction of EBNA2 expression in these cells by estradiol for three days as in [41] did not significantly modify SERCA3 expression, indicating that LMP-1 alone is sufficient for the effect.

### The effects of LMP-1 expression on endoplasmic reticulum calcium homeostasis

In order to establish the effects of LMP-1 expression on B cell calcium homeostasis, resting cytosolic calcium concentration and endoplasmic reticulum calcium storage capacity were measured by Indo-I spectrofluorimetry in the BJAB-tTA-LMP-1 cell line before and after induction of LMP-1 expression. In addition, similar experiments were conducted on non-infected BL-30 and BL-41 Burkitt's-lymphoma cell lines in parallel with BL-30-B95–8 and BL-41-B95–8 cells infected with the immortalizing B95.8 EBV strain that expresses LMP-1.

**Figure 3:**
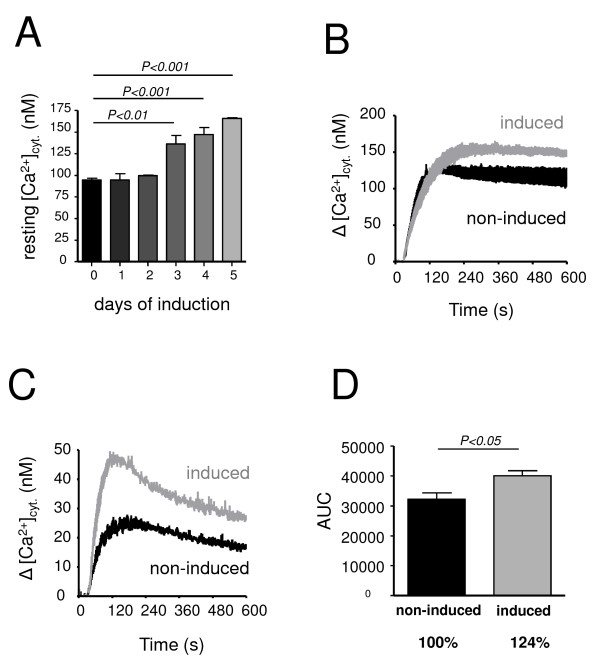
**Modulation of cellular calcium homeostasis by LMP-1 protein expression**. LMP-1 expression in BJAB-tTA-LMP1 cells was induced by tetracycline withdrawal, and resting cytosolic calcium concentration, as well as thapsigargin-induced calcium release from the endoplasmic reticulum were measured. Panel A: The increase of the resting cytosolic calcium concentration in BJAB-tTA-LMP-1 cells during LMP-1 induction. Panels B and C: Calcium release from thapsigargin-sensitive intracellular pools in the presence (Panel B), and in the absence (Panel C) of inhibition of PMCA and Na^+^/Ca^2+ ^exchanger activity. Black curves: non-induced cells, grey curves: cells at day 5 of LMP-1 induction. Panel D: The magnitude of calcium release from non-induced cells (black column) and in cells at day 5 of LMP-1 induction (grey column), expressed as area under curve (AUC) values of measurements presented in Panel B. LMP-1 induction leads to enhanced calcium release from the endoplasmic reticulum by thapsigargin indicating increased calcium sequestration.

**Figure 4:**
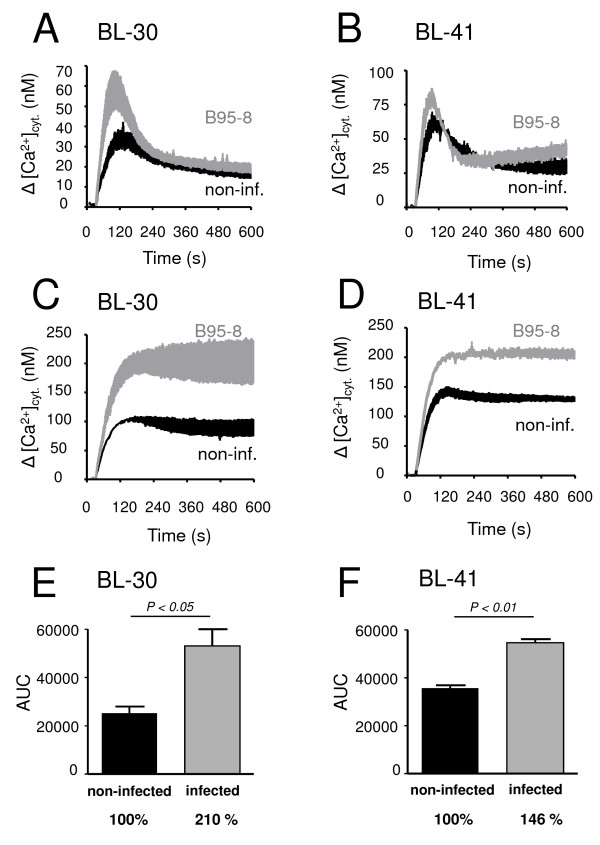
**Enhanced calcium release from the endoplasmic reticulum in EBV-infected cells**. Calcium mobilisation from the thapsigargin-sensitive endoplasmic reticulum calcium pool in non-infected (black curves), and EBV-infected cells (grey curves) was measured in BL-30 (Panels A and C) and BL-41 (Panels B and D) cells. Thapsigargin-induced calcium release from the cells was recorded in the absence (Panels A and B), and in the presence (Panels C and D) of inhibition of PMCA and Na^+^/Ca^2+ ^exchanger-dependent calcium extrusion across the plasma membrane. Panels E and F: Area under curve values of the plateau phase of calcium release obtained in Panels C and D expressed in arbitrary units (AUC). Black columns: non-infected, grey columns: infected cells.

As shown in Fig. 3, induction of LMP-1 expression in the BJAB-tTA-LMP-1 cells led to increased resting cytosolic calcium concentrations (Panel A). Indeed, whereas the resting cytosolic calcium concentration was 95 +/- 2 nM (n = 5) in non-induced cells, LMP-1 induction led to a gradual increase to 165 +/- 1 nM (n = 5) during a five days period. LMP-1 induction was also accompanied by increased calcium release from the ER upon full inhibition of SERCA-dependent transport by 1 μM thapsigargin. As shown in Panel B, when calcium release from the ER was induced by 1 μM thapsigargin in conditions where Ca^2+ ^is trapped in the cytosol due to the inhibition of plasma membrane calcium pumps and Na^+^/Ca^2+ ^exchange by 0.4 μM Hg^2+ ^and replacement of extracellular Na^+ ^by Li^+^, respectively, LMP-1 expressing cells displayed significantly enhanced calcium release from the ER, as reflected by a higher plateau of cytosolic Ca^2+ ^levels (157 +/- 7 nM, n = 5), in LMP-1 expressing cells (Panel B), when compared to non-induced cells (129 +/- 7 nM, n = 5). When expressed as area under curve (AUC) values, LMP-1 expression under these conditions led to an approximately 24% increased calcium release when compared to non-induced cells (Panel D). Increased calcium mobilization from the ER by thapsigargin could also be seen in LMP-1-expressing cells in the presence of extracellular Na^+ ^and in the absence of PMCA inhibition (Panel C), indicating that the observed LMP-1-dependent changes of cellular calcium homeostasis are also present in the presence of active plasma membrane calcium extrusion. The same experiments conducted on control BJAB-tTA cells treated identically to BJAB-tTA-LMP-1 cells, but devoid of LMP-1 expression disclosed no changes in cellular calcium homeostasis upon treatments (not shown). On the other hand, increased calcium storage in thapsigargin-sensitive intracellular calcium pools could also be observed, very similarly to LMP-1 induction, in cells infected with fully competent virus that expresses LMP-1 protein. Indeed, as shown in Fig. 4, when calcium release from the endoplasmic reticulum was induced by thapsigargin in non-infected BL-30 and BL-41 cells (black curves), and in cells infected with wild-type EBV (grey curves), an enhanced calcium release response could be observed in both cell lines in the presence of the virus. When the experiments were performed in the absence of inhibition of plasma membrane calcium pump and Na^+^/Ca^2+ ^exchanger activity, this could be observed as an increased calcium peak following thapsigargin addition in BL-30 (non-infected *versus *infected: 35 +/- 5 nM and 61 +/- 10 nM, n = 3, p > 0.05), as well as BL-41 cells (non-infected *versus *infected: 68 +/- 5 nM and 82 +/- 5 nM, n = 4, p = 0.056). When the same experiments were performed in the presence of inhibition of PMCA and Na^+^/Ca^2+ ^exchanger function, calcium release from the endoplasmic reticulum occurred as a sustained plateau and was markedly enhanced in infected cells. In fact, whereas in non-infected BL-30 and BL-41 cells calcium levels raised to 106 +/- 2 nM (n = 4) and 139 +/- 5 nM (n = 3), respectively, in the corresponding EBV-infected cells calcium levels attained 234 +/- 17 nM (n = 4) and 204 +/- 6 nM (n = 3), respectively, corresponding to a highly significant (p < 0.01) increase in infected cells. When this calcium release was expressed as area under curve (AUC) values of the plateau phase of the corresponding calcium fluorimetry curves of non-infected and B95–8-infected BL-30 and BL-41 cells, a highly significant increase of 110% and of 46% could be observed, respectively (Panels E and F).

### Decreased SERCA3 expression during normal B cell activation *in situ*

**Figure 5:**
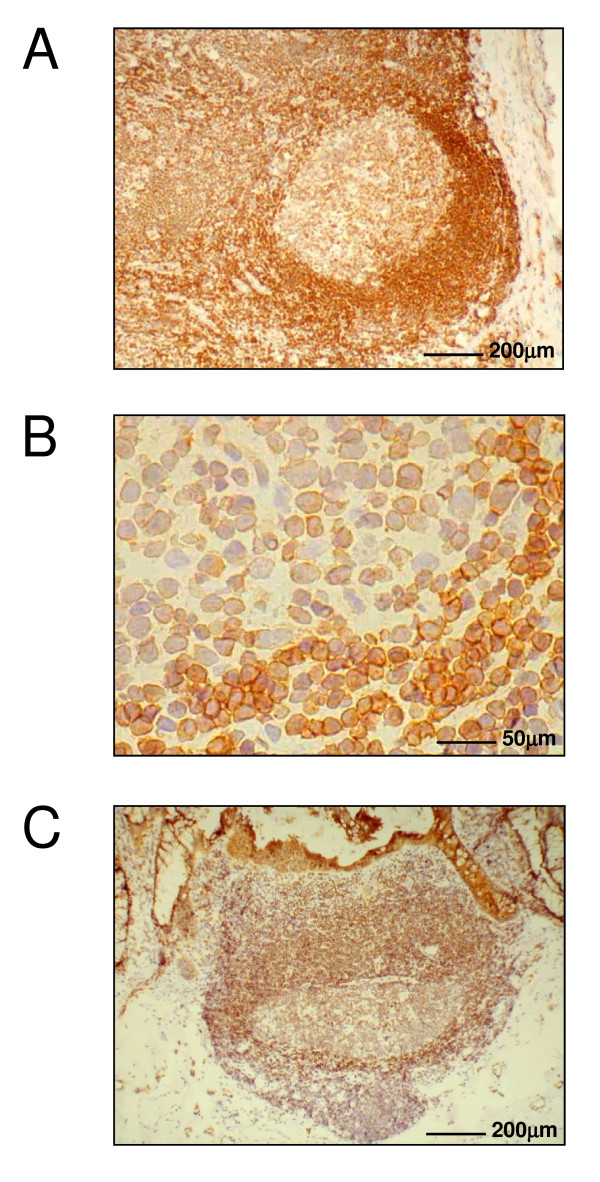
**Immunohistochemical staining for SERCA3 protein in normal resting and activated B lymphocytes**. SERCA expression in lymph node and mucosa associated lymphoid tissue was detected by imunohistochemistry using the PLIM430 monoclonal anti-SERCA3 antibody with o-diaminobenzidine as a chromogen and hematoxylin counterstain. Panel A: lymph node follicle Panel B: mantle zone small resting B lymphocytes (lower right) and germinal center activated lymphoblasts (upper left). Panel C: colonic intraepithelial lymphoid follicle with germinal center.

EBV infection of B cells leads to the emergence of immortalized cell lines that actively proliferate and display a lymphoblastoid phenotype. In order to investigate, whether SERCA3 expression is modulated, similarly to EBV infection, also during normal B lymphocyte activation in a physiological tissue environment *in situ*, immunohistochemical staining for the protein was performed in normal lymph node and colic mucosa-associated lymphoid follicles. In these tissues quiescent, antigen-naïve non-stimulated small B lymphocytes reside in the mantle zone, whereas antigen-activated large centroblasts and centrocytes are located in germinal centers. Such a differential localization of resting and antigen-activated B cell populations in these tissues permits their identification on a histological basis. As shown in Fig. 5, Panel A, SERCA3 expression in a lymph node follicle displays marked differences: whereas the mantle zone that surrounds germinal centers is strongly labelled, staining in the germinal center is markedly weaker. As shown at higher magnification on Panel B, whereas small lymphocytes residing in the mantle zone are strongly labelled, SERCA3 expression in the germinal center activated lymphoblasts is markedly decreased.

In addition to a strong SERCA3 staining in colon epithelium as reported earlier [11,14], a staining pattern very similar to that observed in lymph node follicles could be seen also in mucosa-associated lymphoid tissue in the colon (Panel C), with a marked decrease of SERCA3 expression in germinal centers when compared to small resting B cells located in a more luminal position.

These data taken together indicate that antigen-driven activation of small resting B lymphocytes located in the mantle zone into germinal center lymphoblasts is accompanied by a marked and homogeneous decrease of SERCA3 expression.

## Discussion

The EBV life cycle in B cells is critically regulated by cellular calcium signalling: protein kinase C and calmodulin dependent kinase activation are key factors that determine the transition from latency to virus replication and host cell lysis. In experimental settings this is reflected by the strong capacity of agents that increase cytosolic calcium levels to induce the lytic cycle in latently infected B cells. Indeed, calcium ionophores, such as A23187 or ionomycin, are strong inducers of EBV reactivation [42].

Endoplasmic reticulum calcium homeostasis is also involved in several mechanisms essential for normal B lymphocyte function at various stages of differentiation. Appropriate calcium signalling is necessary for pre-B cell receptor, as well as B-cell receptor-dependent intracellular signalling and lymphocyte selection/survival, differentiation and antigen-dependent activation [27,28,43]. In addition, plasmocytes, that are fully differentiated, antibody secreting B cells, build up large amounts of endoplasmic reticulum, as this organelle is involved in immunoglobulin synthesis and maturation [44-46].

Although the importance of ER calcium homeostasis and signalling in the control of EBV replication is clearly established in the literature [42,47], data on the effect of EBV on ER calcium homeostasis, and in particular on SERCA expression and function were lacking. In this work we show, for the first time, that SERCA expression and activity are modified by EBV in B cells in type III latency by LMP-1. A marked decrease of SERCA3 expression could be observed in various Burkitt's lymphoma cell lines infected with the B95–8 EBV strain that displays full immortalizing capacity and expresses LMP-1 and EBNA2, whereas the non-immortalizing P3HR-1 virus strain that lacks LMP-1 expression due to deletions in the sequence of the EBNA2 viral transactivator [38] was without effect. Importantly, down-modulation of SERCA3 expression could also be induced by the expression of LMP-1 *in trans*, in the absence of any other viral elements, such as EBNA2 or EBNA3A, 3B or 3C, in various inducible expression systems, indicating that LMP-1 alone is sufficient for SERCA3 down-modulation. LMP-1 expression led to increased resting cytosolic calcium levels in accordance with previous observations [48] and, importantly, to increased calcium storage in the ER as detected by thapsigargin-induced calcium release, indicating that LMP-1 can induce functional changes in the ER calcium homeostasis of the host cell. Unlike during induction of the expression of LMP-1 cDNA, resting cytosolic calcium levels were not significantly increased in chronically EBV-infected cells, probably due to secondary compensatory mechanisms, such as increased SERCA2 expression, that occur during the establishment of latency, or due LMP-2A that has been shown to blunt calcium mobilisation in B cells [49]. However, SERCA3 down-modulation and enhanced ER calcium storage could be observed, both in Burkitt's lymphoma cell lines infected with the prototypical, immortalizing B95–8 EBV strain and in EBV-free cells expressing only an LMP-1 transgene. These data taken together show, that EBV modifies SERCA expression as well as functional calcium accumulation into the ER of latently infected B cells, by the LMP-1 viral oncoprotein. LMP-1 activates several signalling pathways including NF-κB, STATs, ERK, JNK, p38 MAPK, as well as PI3K/Akt. Due to the simultaneous activation and cross-talk among these regulatory mechanisms, the identification of the direct molecular mechanism of LMP-1-induced SERCA3 down-regulation requires further work. However, our data show, for the first time, that the modulation of ER calcium homeostasis is part of the cell activation program induced by LMP-1.

The functional consequences of the modulation of SERCA expression by EBV in B cells are complex. The various protein types involved in cellular calcium homeostasis function in a highly interconnected manner, due to the fact that the transport activity of various SERCA and PMCA enzymes, as well as the opening probability of IP3 receptor calcium channels are regulated by the calcium concentration of the cytosol or of the ER lumen. This leads to positive, as well as negative feedback regulation that often generate oscillatory cellular calcium signals, the frequency and amplitude of which can selectively affect the activation of target proteins such as PKC or calcineurin [50-54]. Computer modelling of the highly dynamic interplay of calcium pumps, channels and target enzymes, coupled to experimental observation made on cells in which SERCA3 expression was ablated, led to the conclusion that SERCA3 function is particularly important for the shaping of the characteristics (amplitude, frequency and duration) of calcium oscillations [31-33,55,56]. For instance, it has been shown, that the loss of SERCA3 expression leads to altered glucose-induced calcium oscillations and electrical activity that control insulin secretion in the β cells of the endocrine pancreas [55,57]. The calcium affinity of SERCA3 (KCa^2+^ ~ 1.2 μM) is significantly lower than that of the simultaneously expressed SERCA2b isoenzyme (KCa^2+^ ~0.2 μM) [19,21,22,58]. Because the variations of cytosolic calcium levels during cell activation in the vicinity of the ER lie within the range in which SERCA3 activity is modulated by calcium, the amount of SERCA3 expressed in a cell has a major impact on the shape and frequency of calcium signals, and therefore can determine the state of activation of the cell, whereas SERCA2b function is more probably involved in constitutive calcium uptake in the ER, linked to "housekeeping" functions, as this enzyme is probably almost fully active already at resting cytosolic calcium levels.

The results presented in this paper on increased intracellular calcium pool size in the presence of EBV or LMP-1 expression are compatible with the observed decrease of SERCA3 expression. It may be hypothesised that decreased SERCA3 expression reflects the loss or depletion of a lower affinity or leakier intracellular ER sub-compartment, leading to calcium accumulation into a higher affinity, SERCA2b associated ER pool containing more calcium releasable by SERCA inhibition. The exact structural and functional relationship between SERCA2 and SERCA3-associated intracellular calcium pools in B cells remains, however, to be determined.

Enhanced calcium accumulation induced by LMP-1 may be involved in the establishment of viral latency. Because the EBV lytic cycle can be induced by increased cytosolic calcium levels (as observed with calcium ionophores or during B-cell receptor activation [42,47,59]), a more stringent calcium sequestration in the ER by the higher affinity SERCA2b isoform may blunt calcium signals that would otherwise lead to the induction of the lytic cycle. Moreover, SERCA3 has been shown earlier to be associated with the IP3-sensitive sub-compartment of the ER in platelets [60]. It is therefore tempting to hypothesize that the selective down-regulation of SERCA3 expression in EBV infected B cells may correspond to decreased calcium uptake in the IP3-sensitive intracellular calcium pool with calcium uptake being redirected towards a SERCA2-dependent sub-compartment of the ER. This may lead to a state of chronic, constitutive activation of the cells and their desensitisation for further IP3-mobilizing signals. Although the description of the detailed mechanisms of the involvement of ER calcium homeostasis in EBV replication and latency require further studies, our work shows, for the first time, that ER calcium homeostasis is modulated by EBV *via *LMP-1, and opens new avenues in the understanding of the interactions taking place between EBV and the infected lymphocyte.

SERCA3 down-modulation was observed also during normal B lymphocyte activation in lymph node, as well as in mucosa-associated lymphoid follicles. In these tissues the mantle zone represents small resting B lymphocytes admixed with sparse helper and suppressor T cells, whereas in germinal centers where antigen stimulation of B cells by follicular dendritic cells takes place, centrocytes and centroblasts accumulate [43]. As shown in this work by immunohistochemistry, whereas small resting B lymphocytes in the mantle zone express SERCA3 abundantly, SERCA3 expression markedly decreases during antigenic stimulation in germinal centers, indicating that SERCA3 down-modulation is part of the normal B lymphocyte activation program, where this phenomenon is related to antigen-driven activation and blastic transformation of B cells. Although EBV-immortalized lymphoblastoid cells and normal germinal center B lymphoblast display phenotypic differences, both cell types are activated, proliferating B cells. Down-modulation of SERCA3 expression by EBV should be considered therefore as a previously unknown virus-induced phenomenon taking place during establishment of type III latency and immortalization that mimics a normal step of B cell activation.

## Conclusion

The expression of SERCA3 has recently been shown to be modulated during differentiation in cells of colon and gastric carcinoma, as well as various types of myeloid leukaemia [11,12,14,15,61,62]. These observations indicate that SERCA3 function is modified during the differentiation process of various cell types. Interestingly, the selective down-modulation of SERCA3 expression has been observed earlier during T lymphocyte activation [16]. When taken together with data shown in the present work, this indicates, that the down-modulation of SERCA3 expression is a general phenomenon during T, as well as B lymphocyte activation, and that ER calcium homeostasis is remodelled during viral, as well as antigen-driven or pharmacologically induced activation of both lymphoid lineages. In addition to its interest for a more detailed understanding of the biology of EBV-induced B cell immortalisation, our data suggest, that ER calcium homeostasis may constitute a new potential target for the modulation of virus-induced activation and immortalization of B cells by, for example, the direct pharmacological modulation of SERCA activity. In addition, SERCA3 may be useful as a new phenotypic marker for the detection of the acquisition of an activated lymphocyte phenotype during EBV-induced immortalization. As SERCA3 down-modulation is downstream of functionally active LMP-1, SERCA3 immunohistochemistry may also be a useful tool to characterize lymphomas by reflecting the involvement of LMP-1 in the process of tumorigenesis.

## Methods

### Cell culture

Cells were grown at 37°C in a humidified cell culture incubator in an atmosphere of air containing 5% CO_2 _in RPMI-1640 medium supplemented with 2 mM glutamine, 2 mM alanyl-L-glutamine and 10% heat-inactivated fetal calf serum (complete RPMI medium). Cell culture reagents were from Lonza, Verviers, Belgium.

### Induction of LMP-1 expression by tetracycline withdrawal

BJAB-tTA-LMP-1 cells were grown in complete RPMI medium supplemented with 2 mg/ml G418 and 0.5 mg/ml hygromycin B (both purchased from Sigma-Aldrich France) and 1 μg/ml tetracycline (Fluka, Germany) as described earlier [39]. Exponentially growing cells cultured in the presence of 1 μg/ml tetracycline were washed as follows: after centrifugation the cell pellet was resuspended in 10 ml complete medium containing 10% fetal calf serum without tetracycline, transferred into a new 50 ml tube, supplemented with 35 ml serum-free RPMI-1640 medium and centrifuged again. This washing step was repeated three times. Thereafter cells were resuspended in complete RPMI culture medium without tetracycline at an initial density of 2 × 10^5 ^cells/ml. During the last one or two passages preceding induction of LMP-1 expression and during induction by tetracycline withdrawal, selection antibiotics were omitted.

### Induction of LMP-1 expression by zinc

BL41-MTLM-2 cells [40] grown in complete RPMI medium in the exponential phase of growth were centrifuged and resuspended at an initial density of 3 × 10^5 ^cells/ml in complete RPMI medium. After one hour pre-incubation at 37°C in a cell culture incubator, 100 μM ZnCl_2 _(Sigma-Aldrich France, Saint-Quentin Fallavier) was added from a 100-fold concentrated sterile-filtered stock solution. EBNA2 expression was induced in BL41/K3 cells that carry an estradiol-inducible EBNA2 construct as described in [41].

### Sample preparation and Western blotting

This was done essentially as described in [11]. Briefly, after treatments cells were harvested by centrifugation, resuspended in 1 ml ice cold NaCl (150 mM) and transferred to round-bottom 2 ml Eppendorf tubes, centrifuged and washed again by centrifugation with 1 ml ice cold NaCl solution. Cell pellet was thereafter resuspended in ice cold 5% trichloroacetic acid and kept at 4°C overnight. The protein precipitate was centrifuged at 12000 × g for 10 minutes at 4°C and supernatant was removed. The protein pellet was thereafter dissolved in lysis buffer at 30 mg TCA-precipitated protein pellet/ml lysis buffer on a horizontal shaking platform and thereafter stored at -80°C. SDS-polyacrylamide gel electrophoresis (60 μg total cellular protein per well) in 8% (for SERCA pumps) and 10% (for LMP-1) gels and transfer onto nitrocellulose membranes was done as described in [11,14]. The presence of equal amounts of total protein per lane was controlled by Ponceau S staining and densitometry as described previously [11,14]. SERCA2 and SERCA3 expression was detected using the IID8 and the PLIM430 antibodies, respectively, as described in detail [11,14,15]. Luminograms were obtained using the Enhanced Chemiluminescence (ECL) reagent kit of Amersham and were quantitated with the ScionImage software (Scion Corp. CA). Acquisition and quantitative analysis of SERCA signals by Western blotting has been described earlier in detail [11,14,15]. LMP-1 was detected similarly by Western blotting using an anti-LMP monoclonal antibody cocktail (Clones CS.1–4, Code N° M 0897) of DakoCytomation, Denmark.

### Immunohistochemistry

Detection of SERCA3 protein in tissues was performed exactly as described in detail [11]. Briefly, after rehydration 5 μm thick acetone fixed frozen tissue sections were incubated with Tris-buffered saline supplemented with 5% non-fat milk powder and 0.1% Tween-20 (blocking solution) to inhibit non-specific protein binding, followed by incubation with the PLIM430 anti-SERCA3 monoclonal antibody (1 μg/ml) in the same solution. After two cycles of washing with water and blocking solution, antigen binding was detected using biotinylated anti-mouse IgG antibody and avidin-biotin-peroxydase complex (Vectastain ABC kit; Vector Laboratories, Burlingame, CA). Signal was revealed with 3,3'-diaminobenzidine as chromogen, and slides were counterstained with hematoxylin. Omission of the PLIM430 primary antibody, replacement by isotype-matched irrelevant antibody or normal mouse IgG were used as negative controls, and these gave no staining. Strong positive staining of vascular endothelial cells, present in the sections and known to express SERCA3 [21,63-66] served as internal positive control.

Immunocytochemistry for LMP-1 expression was performed on BJAB-tTA-LMP-1 cells before and after induction of LMP-1 expression by tetracycline withdrawal. Cells were washed with PBS by centrifugation, resuspended in PBS and cell smears were made on poly-lysine coated microscope slides and dried on air overnight. After fixation in acetone (10 min. at room temperature), slides were rehydrated and stained with a cocktail of anti-LMP monoclonal antibodies (Clones CS.1–4, Code N° M 0897, DakoCytomation, Denmark) according to the instructions of the manufacturer, using an ABC avidin-biotin-peroxydase system with 3,3'-diaminobenzidine as chromogen, and were counterstained with hematoxilin. Photographs of cells were obtained using a Leica microscope and a Nikon Coolpix 950 digital photographic camera.

### Calcium fluorimetry

Cytosolic ionized free calcium concentration ([Ca^2+^]_cyt_) was measured by a fluorimetric ratio technique [67]. Cells were centrifuged and resuspended at a density of 10^6 ^cells/ml in phosphate-buffered saline (PBS) supplemented with 1 mg/ml bovine serum albumin and incubated in the dark with 4 μM Indo-1 AM for one hour at room temperature under slow agitation. Cells were then centrifuged and resuspended in calcium-free Hanks' buffered saline solution (HBS; 135 mM NaCl, 5.9 mM KCl, 1.2 mM MgCl_2_, 11.6 mM Hepes, 11.5 mM glucose adjusted to pH 7.3 with NaOH) prior measurements. After centrifugation, 0.5 to 1 × 10^6 ^cells were suspended in 3 ml HBS in a quartz cuvette and inserted into a Shimadzu RF1501 spectrofluorimeter equipped with a stirring apparatus and a thermostatted (37°C) cuvette holder, and connected to a PC computer. Ultraviolet light at 360 nm was used for excitation of Indo-1, and emission at 405 and 480 nm was recorded in time before and after addition of reagents as indicated in figures. Background and autofluorescence of the cell suspensions were subtracted from the recordings. Maximum Indo-1 fluorescence (F_max_) was obtained by adding 1 μM ionomycin to the cell suspension in the presence of 10 mM CaCl_2_, and minimum fluorescence (F_min_) was determined without added calcium in the presence of 5 mM EGTA. Measurements of resting cytosolic calcium concentrations in the presence of extracellular calcium were performed in HBS supplemented with 1 mM CaCl_2_. Cytosolic calcium concentrations were calculated according to the equation [Ca^2+^]_cyt _= Kd(R-R_min_)/R_max_-R), where Kd is the apparent dissociation constant of the Indo-1-calcium complex (230 nM), and R is the ratio of fluorescence values (F) measured at 405 and 480 nm (R = F_405_/F_480_).

In appropriate experiments, inhibition of the Na^+^/Ca^2+ ^exchangers was obtained by replacement of all Na^+ ^by Li^+ ^in the HBS bath solution, and plasma membrane calcium pump activity was inhibited by 0.4 μM HgCl_2 _[68].

### Statistical analysis

Data are presented as the mean +/- SEM and correspond to at least three independent experiments; the number of experiments performed is indicated in brackets. Statistical analysis was performed using Student's paired t-test.

## Competing interests

The authors declare that they have no competing interests.

## Authors' contributions

OD and BP designed and performed research, collected, analysed and interpreted data and wrote the manuscript. CC interpreted data, AA performed research, collected and analyzed data, IJ, JPB, MR and TK contributed vital reagents and interpreted data. All authors read and approved the final manuscript.
